# Pharmacokinetics of Sulfasalazine and the Temporal Profiles of Sulfapyridine and Its Metabolites Following Nasogastric Administration in Six Healthy Adult Horses

**DOI:** 10.3390/ani16213530

**Published:** 2026-11-07

**Authors:** Miłosz Grabski, Olga Wachełko, Marcin Zawadzki, Paweł Szpot, Ewa Kumiega, Artur Niedźwiedź

**Affiliations:** 1 Equi Vet Serwis, Wygoda 6, 64-320 Buk, Poland; 2 Faculty of Medicine, Wroclaw University of Science and Technology, 27 Wybrzeże Wyspiańskiego, 50-370 Wrocław, Poland; olga.wachelko@iet.wroc.pl; 3 Department of Social Sciences and Infectious Diseases, Faculty of Medicine, Wroclaw University of Science and Technology, 27 Wybrzeże Wyspiańskiego, 50-370 Wrocław, Poland; m.zawadzki@pwr.edu.pl; 4 Department of Forensic Medicine, Wroclaw Medical University, 4 J. Mikulicza-Radeckiego Street, 50-345 Wroclaw, Poland; pawel.szpot@iet.wroc.pl; 5 Department of Internal Medicine with Horse, Dog and Cat Clinic, Faculty of Veterinary Medicine, Wroclaw University of Environmental and Life Sciences, pl. Grunwaldzki 47, 50-366 Wrocław, Poland; ewa.kumiega@upwr.edu.pl (E.K.); artur.niedzwiedz@upwr.edu.pl (A.N.)

**Keywords:** horse, sulfasalazine, sulfapyridine, pharmacokinetics, prodrug, hindgut microbiota

## Abstract

Sulfasalazine is an azo-linked prodrug of sulfapyridine and 5-aminosalicylic acid that is activated by bacterial azoreductases in the distal gut. Its disposition has not been described in horses, in which extensive hindgut fermentation may influence prodrug activation. This study characterised the concentration-time profile of sulfasalazine and the appearance of sulfapyridine, 5-hydroxy-sulfapyridine and N^4^-acetyl-sulfapyridine after a single oral dose. Six healthy adult Warmblood horses received sulfasalazine (16 mg/kg) by nasogastric tube, and blood was sampled hourly for 14 h. Sulfasalazine and sulfapyridine were quantified by liquid chromatography-tandem mass spectrometry; both metabolites were assessed semi-quantitatively as peak-area ratios. Sulfasalazine was measurable from 1 h and reached a median maximum concentration of 386.61 ng/mL (Q1–Q3 330.44–504.35) at a median of 5.5 h, then declined steeply. Sulfapyridine remained near the limit of detection for six hours, rose abruptly between 7 and 8 h and peaked at a median of 2794.00 ng/mL (2381.87–3146.14) at 9.0 h, approximately seven-fold above the parent-drug maximum. Both metabolites rose in parallel with sulfapyridine. This timing matches published equine orocaecal transit times, indicating that sulfasalazine reaches the hindgut largely intact and is activated there by microbial azoreduction. Systemic sulfapyridine exposure was substantial and should be considered in any therapeutic use.

## Simple Summary

Sulfasalazine is an inexpensive anti-inflammatory drug used for long-term bowel inflammation in people and dogs. It is given in an inactive form and only becomes active when bacteria in the large intestine split it into its two components. Horses also develop chronic inflammatory bowel disease, for which treatment options are limited, but how sulfasalazine behaves in horses has never been studied. We gave a single dose of sulfasalazine by stomach tube to six healthy horses and measured the drug and its breakdown products in their blood every hour for 14 h. Only small amounts of the intact drug reached the bloodstream. Its main breakdown product, sulfapyridine, appeared suddenly seven to eight hours after dosing, which matches the time food takes to reach the horse’s large intestine. This indicates that the drug passes through the stomach and small intestine largely unchanged and is activated by bacteria in the hindgut, as intended. Blood levels of sulfapyridine were, however, much higher than those of the original drug, which matters for possible side effects. These findings provide the first basis for studying sulfasalazine as a treatment for intestinal inflammation in horses.

## 1. Introduction

Sulfasalazine (salicylazosulfapyridine) is a prodrug in which sulfapyridine and 5-aminosalicylic acid (5-ASA, mesalazine) are joined by an azo bond [1]. Developed in the 1940s, it remains a first line treatment for mild to moderate ulcerative colitis and a disease modifying drug in rheumatoid arthritis in human medicine [1,2], and is used for chronic inflammatory enteropathies in dogs [1,3].

The pharmacology of sulfasalazine is determined largely by the fate of its azo bond. After oral administration only a small proportion of the dose (approximately 3–15% in humans) is absorbed intact from the proximal gastrointestinal tract. The remainder passes into the distal bowel, where bacterial azoreductases cleave the azo linkage and release the two constituent moieties [1,4,5,6]. This microbial step is obligatory. In germ-free or antibiotic-treated animals, and in patients after colectomy, the prodrug is excreted largely unchanged [4,5]. Azoreductase activity resides mainly in obligate anaerobes such as *Clostridium*, *Eubacterium*, *Butyrivibrio* and *Bacteroides* spp. [6,7], and because their abundance varies between individuals and between host species, prodrug activation is itself a source of pharmacokinetic variability [8,9]. Released 5-ASA remains largely within the intestinal lumen, where it acts topically [1], whereas sulfapyridine is efficiently absorbed and accounts for essentially all systemic exposure and most adverse effects [1,4]. Sulfapyridine is cleared by hepatic N^4^-acetylation (NAT2) and by ring hydroxylation with glucuronidation [1,10]. In humans, slow acetylators have greater sulfapyridine exposure and a materially higher risk of adverse drug reactions [11,12]. Because sulfapyridine appears systemically only once the prodrug has reached the distal gut, the interval between dosing and its appearance in plasma or saliva has been validated as a non-invasive measure of orocaecal transit time in humans [13,14] and dogs [15].

There is a clear clinical rationale for evaluating sulfasalazine in horses. Chronic inflammatory bowel disease (IBD) in this species comprises a group of idiopathic infiltrative enteropathies, including granulomatous enteritis, lymphocytic-plasmacytic and eosinophilic enterocolitis and multisystemic eosinophilic epitheliotropic disease, that present with weight loss, hypoproteinaemia, diarrhoea or recurrent colic [16,17]. Treatment remains empirical and rests almost entirely on systemic corticosteroids, to which the response is inconsistent and whose prolonged use carries a recognised risk of laminitis and immunosuppression, and the long-term prognosis for most forms is guarded to poor [16,17]. Right dorsal colitis, typically associated with non-steroidal anti-inflammatory drug administration, is likewise managed largely supportively, with few targeted anti-inflammatory options [18,19]. A gut-directed anti-inflammatory agent that is inexpensive, orally administered and established in the corresponding human and canine diseases would therefore fill an evident therapeutic gap. Published clinical experience in horses is, however, limited to a single case report, in which an adult horse with chronic diarrhoea attributed to suspected inflammatory colitis responded to oral sulfasalazine at 16 mg/kg once daily [20]. The drug is also discussed in a recent review of equine inflammatory bowel disease [21] and is used off label in horses for inflammatory large bowel disease [22]. No pharmacokinetic data are available for this species, and a review of right dorsal colitis noted two decades ago that “the effects of sulphasalazine in the horse have not been evaluated” [18].

Extrapolation from other species is not straightforward. The horse is a hindgut fermenter whose caecum and ventral colon contain a dense and metabolically active anaerobic community [23,24,25,26,27]. Passage through the stomach and small intestine takes approximately five hours, orocaecal transit times in healthy horses fall between approximately 5.8 and 8.0 h [28,29,30], and retention within the caecum and colon averages approximately 35 h [31]. An azo-linked prodrug should therefore reach the fermentative compartment substantially intact and remain in prolonged contact with a large microbial population. Whether this prediction holds is unknown. Azoreductase activity has not been demonstrated in equine intestinal contents, and the expectation rests on taxonomic overlap with genera characterised in other hosts [6,23,26]. Oral drug bioavailability in horses ranges from a few percent to near-complete depending on the drug, feeding state and formulation [32,33,34,35,36], and equine hepatic acetylation capacity is low, with hydroxylated rather than N^4^-acetylated sulfonamide metabolites predominating in plasma [37,38,39].

The objective of the present study was therefore to characterise, for the first time, the pharmacokinetics of sulfasalazine and the temporal profiles of sulfapyridine, 5-hydroxy-sulfapyridine and N^4^-acetyl-sulfapyridine following a single oral dose of sulfasalazine in healthy adult horses.

## 2. Materials and Methods

### 2.1. Ethical Approval

The study was reviewed and approved by the Local Ethics Committee for Animal Experiments in Wrocław, Poland (approval number 12/2021, 17 February 2021). All procedures were conducted in accordance with applicable national legislation governing the protection of animals used for scientific purposes. Written informed consent was obtained from the owners of all participating horses.

### 2.2. Animals

The study population comprised six clinically healthy adult Warmblood horses (three geldings and three mares) aged between 5 and 15 years and weighing between 430 and 580 kg. Body weight was estimated using a commercial equine weight tape (HorseGuard; Unamari, Haderslev, Denmark). Data from one further horse, sampled under the same protocol, were excluded from all analyses because the internal standard failed during analysis of its samples, rendering its results unreliable.

Health status was established by physical examination, including assessment of demeanour, rectal temperature, heart and respiratory rates, mucous membrane appearance, capillary refill time, gastrointestinal borborygmi and faecal consistency. All horses were in good body condition, with scores of 5–6 on the 9-point Henneke scale [40]. Exclusion criteria were any history of gastrointestinal disease or signs of abdominal pain within the preceding six months, administration of anti-inflammatory medication during the same period, or any previous administration of sulfonamides. All horses were maintained under a standard anthelmintic programme.

Horses were housed individually in stalls for the duration of the sampling period and remained under continuous observation. Water was available ad libitum throughout. Feeding was standardised across animals: horses were fasted for 12 h before dosing and were offered mixed-grass meadow hay (predominantly Poaceae species) only from 6 h after administration. The hay was typical of forage produced from permanent grasslands in Poland and was thereafter fed at a maintenance ration of approximately 10–12 kg per 500 kg body weight per day. This regime, which differs substantially from routine management, was chosen to standardise gastric contents at the time of dosing; its likely influence on gastrointestinal transit is considered in Section 4.5.

### 2.3. Study Design and Drug Administration

This was a prospective, single-dose, open label pharmacokinetic study. Each horse received a single dose of sulfasalazine at 16 mg/kg body weight. The dose of 16 mg/kg was selected because it is the empirical dose listed for horses in a standard veterinary formulary [41] and the initial dose used in the only published equine clinical report [20], no pharmacokinetic data being available to guide dose selection in this species, and because it falls within the per-administration range used in dogs (10–30 mg/kg every 8–12 h) [3,42]. The dose was prepared from commercially available 500 mg sulfasalazine tablets (Krka, Novo mesto, Slovenia), with the number of tablets rounded to the nearest whole tablet according to the estimated body weight. The tablets were crushed, suspended in 50 mL of water, and administered directly into the stomach via a nasogastric tube. The tube was subsequently flushed with a further 500 mL of water to ensure complete delivery of the dose before being withdrawn.

Horses were observed continuously throughout the sampling period and monitored for 36 h thereafter for adverse effects, including signs of abdominal discomfort, altered faecal consistency, and changes in demeanour or appetite. Rectal temperature was measured before drug administration and after collection of the final blood sample.

### 2.4. Blood Sampling and Sample Handling

Blood samples were collected hourly from 1 to 14 h after administration, resulting in 14 samples per horse and 84 samples in total. No pre-dose sample was obtained; the concentration at time zero was therefore assumed to be zero on the basis of the exclusion of any previous sulfonamide administration (Section 2.2). Samples were obtained via an indwelling jugular catheter, with the first 10 mL discarded before each collection.

Blood was collected into serum separator tubes, allowed to clot at room temperature for 45 min where applicable, and centrifuged at 1500× *g* for 10 min at 20 °C. The resulting serum was transferred to cryovials and stored at −80 °C until analysis.

### 2.5. Analytical Procedures

Sulfasalazine and sulfapyridine were quantified using a validated ultra-high performance liquid chromatography tandem mass spectrometry (UHPLC-MS/MS) method. Samples were prepared by liquid-liquid extraction with ethyl acetate at pH 9, with sulfasalazine-d4 added before extraction, following a previously described sample preparation procedure [43]. Chromatographic separation was achieved using a UPLC BEH C18 column (100 × 2.1 mm i.d., particle size 1.7 μm; Waters, Milford, MA, USA) with a 0.1% formic acid and 10 mM ammonium formate in water (A) and 0.1% formic acid and 10 mM ammonium formate in methanol (B) as mobile phases and gradient at a flow rate of 0.4 mL/min. Detection was performed in positive electrospray ionisation mode using multiple reaction monitoring, with transitions of *m*/*z* 399.0→381.1 (quantifier ion), 223.2, 119.1 for sulfasalazine; 250.0→108.1 (quantifier ion), 156.1, 91.95 for sulfapyridine; and 403.0→384.95 (quantifier ion), 222.95, 119.2 for the internal standard.

The method was validated for selectivity, linearity, accuracy, precision, recovery, matrix effect and stability in accordance with GTFCh bioanalytical method validation guidance. Calibration curves were linear in the range of 1–2000 ng/mL for both analytes (r^2^ ≥ 0.997). The lower limit of quantification (LLOQ) was 1 ng/mL for both analytes. Intra- and inter-day accuracy were within ±14.5%, and precision, expressed as coefficient of variation, was below 10.5% at all quality-control concentrations. Samples with sulfapyridine concentrations exceeding the upper limit of quantification were diluted 10-fold with blank matrix and reanalysed. The dilution integrity was confirmed at the same concentration level. Analytical batches included double blank, blank plus internal standard, QC at the concentration of 10 ng/mL and carryover samples. No carryover was observed. Concentrations below the LLOQ could not be quantified and are reported as <LLOQ; for the calculation of summary statistics and the area under the curve, the values estimated from the calibration curve were retained. Setting these values to zero instead changed AUC_0–14h_ by less than 0.01% for both analytes.

Certified reference standards for 5-hydroxy-sulfapyridine and N^4^-acetyl-sulfapyridine were not available; these metabolites were therefore evaluated semi-quantitatively. Results are expressed as the ratio of analyte peak area to internal-standard peak area (Area/Area_IS_), providing a dimensionless index that allows comparison of relative signal intensity across time points within a single analyte. Because the ionisation efficiency of the two metabolites differs and no response factors were determined, these values do not represent absolute concentrations, cannot be compared between the two metabolites, and cannot be directly compared with values reported elsewhere.

### 2.6. Pharmacokinetic and Statistical Analysis

Pharmacokinetic parameters were derived from individual concentration time profiles using non-compartmental analysis in Statistica 13 (TIBCO Software Inc., Palo Alto, CA, USA). Maximum observed concentration (C_max_) and the corresponding time (T_max_) were obtained directly from the observed data for each horse without interpolation. The area under the concentration time curve from administration to the final sampling point (AUC_0–14h_) was calculated using the linear trapezoidal method, with the concentration at time zero assumed to be zero.

Because the sampling period extended only to 14 h, AUC is reported as the truncated AUC_0–14h_. AUC extrapolated to infinity was not calculated because the terminal log-linear phase was not adequately characterised for all analytes, and extrapolation from this sampling window would not satisfy the conventional requirement that the observed AUC account for at least 80% of AUC_0–∞_ [44,45]. For the semi-quantitative metabolites, the corresponding measure is the area under the ratio-time curve and is expressed in hours.

The study was designed as a descriptive characterisation, and the analysis is correspondingly descriptive. No hypothesis testing between groups or treatments was undertaken, so the sample size was not based on a formal power calculation; six animals is consistent with the group sizes typically used in exploratory pharmacokinetic studies in horses, and the results are intended to inform the design of adequately powered subsequent studies. Data distribution was assessed using the Shapiro-Wilk test; given the small sample size this test has limited power, and nonparametric summary statistics were adopted primarily because the distribution of concentrations at several time points was visibly skewed and constrained by the limit of quantification. Results are therefore presented as median with first and third quartiles (Q1–Q3) and, in the figures, with minimum and maximum values. Statistical significance was set at α = 0.05. Statistical analyses were performed using Statistica, version 13 (TIBCO Software Inc., Palo Alto, CA, USA).

Figures were drawn in Python 3.11 (Matplotlib 3.8). Boxes show the medians and quartiles reported in Table 1; the whiskers (minimum-maximum values) and the individual profiles in Supplementary Figure S1 were digitised from the original Statistica plots. Digitisation and plotting scripts were prepared with the assistance of a generative AI tool and verified by the authors.

## 3. Results

### 3.1. Protocol Implementation

All six horses received the complete intended dose and completed the sampling protocol. No adverse effects were observed during administration or throughout the sampling period. Rectal temperatures measured before dosing and after the final sample were within physiological limits in all horses. Nasogastric intubation was tolerated without incident in all horses.

### 3.2. Sulfasalazine

**Figure 1:**
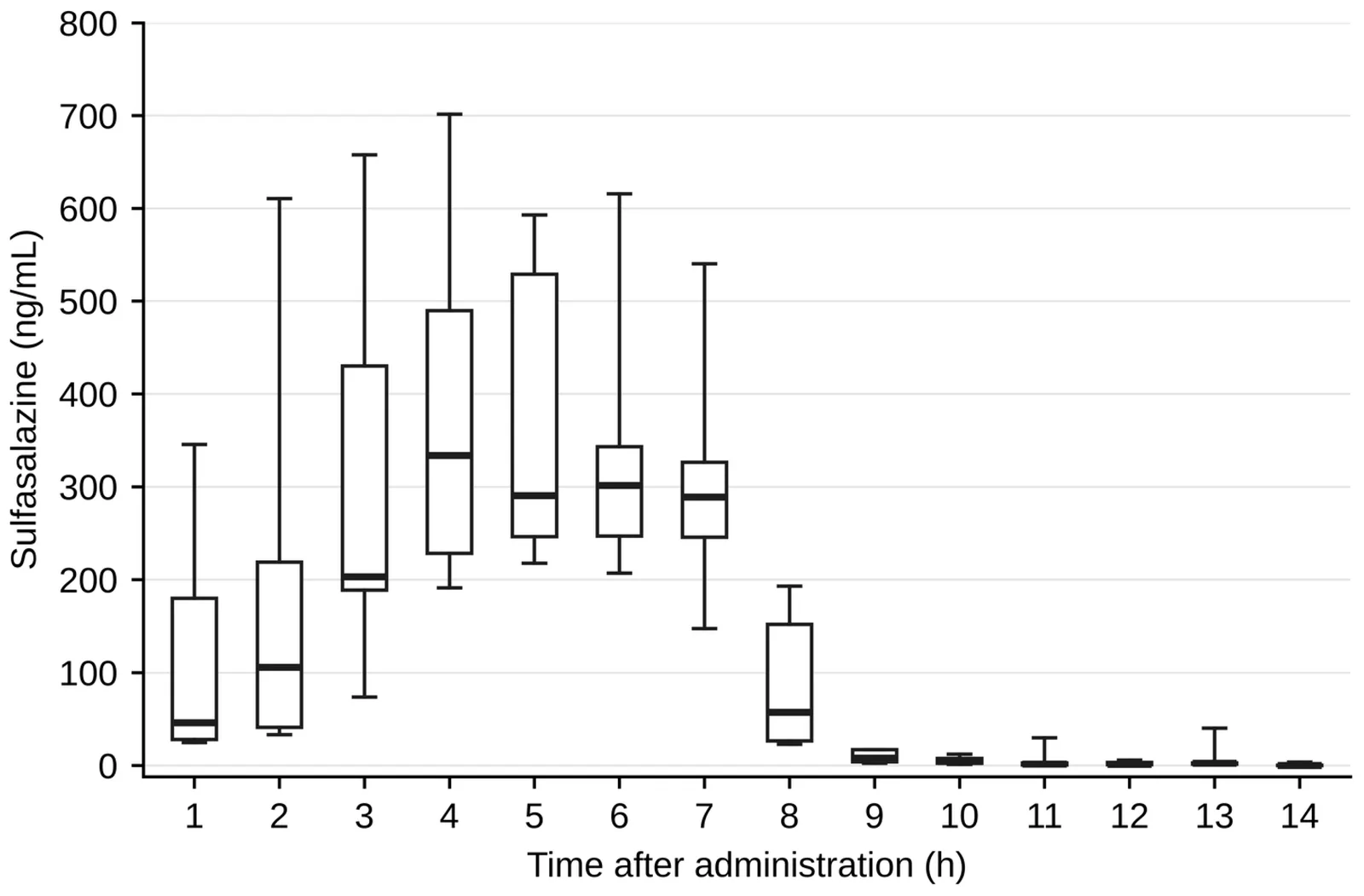
Serum sulfasalazine concentrations in six healthy adult horses at 14 hourly time points following nasogastric administration of sulfasalazine at 16 mg/kg. Data are presented as median (Q1–Q3), with minimum-maximum values.

Sulfasalazine was quantifiable in all horses at the first sampling point. Median concentrations rose over the first 4 h and then remained on a plateau of approximately 290–330 ng/mL until 7 h. Between 7 and 8 h the median fell approximately five-fold, and it declined steeply thereafter to values at or close to the lower limit of quantification from 11 h onward; small fluctuations at 12–14 h should be regarded as analytical noise rather than as a meaningful feature of the profile (Table 1; Figure 1).

Individual concentration-time profiles (Supplementary Figure S1) showed the same overall pattern in every horse, with a rise to a peak or plateau followed by a steep fall between 7 and 9 h. Individual peak concentrations were, however, markedly variable, ranging approximately 2.6-fold (from approximately 270 to 700 ng/mL), and individual T_max_ ranged from 3 to 7 h, whereas the timing of the late decline was consistent between animals.

### 3.3. Sulfapyridine

**Figure 2:**
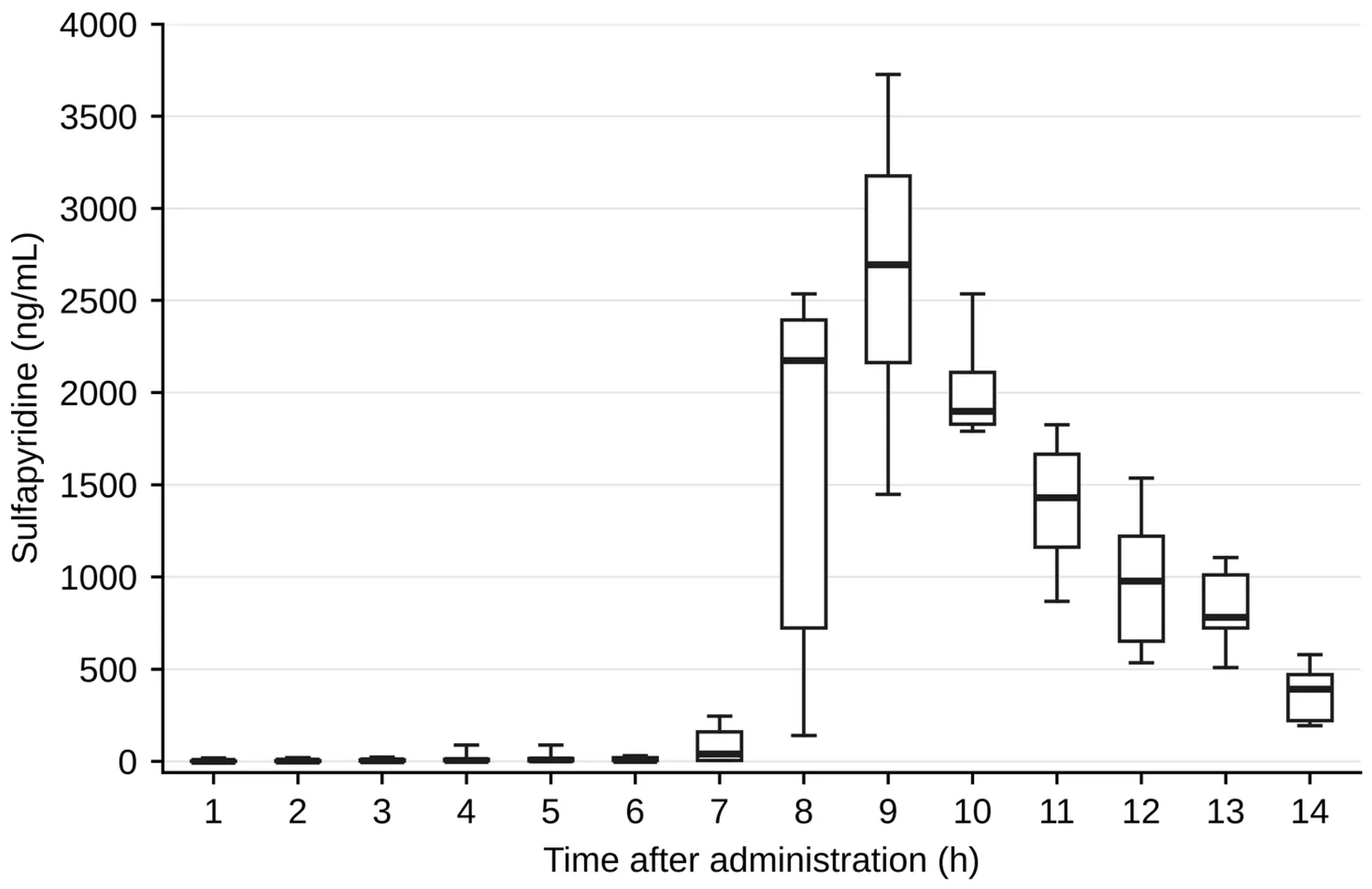
Serum sulfapyridine concentrations in six healthy adult horses at 14 hourly time points following nasogastric administration of sulfasalazine at 16 mg/kg. Data are presented as median (Q1–Q3), with minimum-maximum values.

Sulfapyridine concentrations remained low (median 8.49 ng/mL or less) during the first 6 h, while parent-drug concentrations were highest. The median then rose from 40.03 ng/mL at 7 h to 2173.78 ng/mL at 8 h, an approximately 54-fold increase within a single hour; the wide interquartile range at 7 h indicates that the exact hour of transition varied between animals. Concentrations peaked at 9 h and declined progressively to 390.82 ng/mL at 14 h (Table 1; Figure 2). The rise in sulfapyridine therefore coincided with the fall in sulfasalazine in the 7–8 h interval.

### 3.4. Sulfapyridine Metabolites

**Figure 3:**
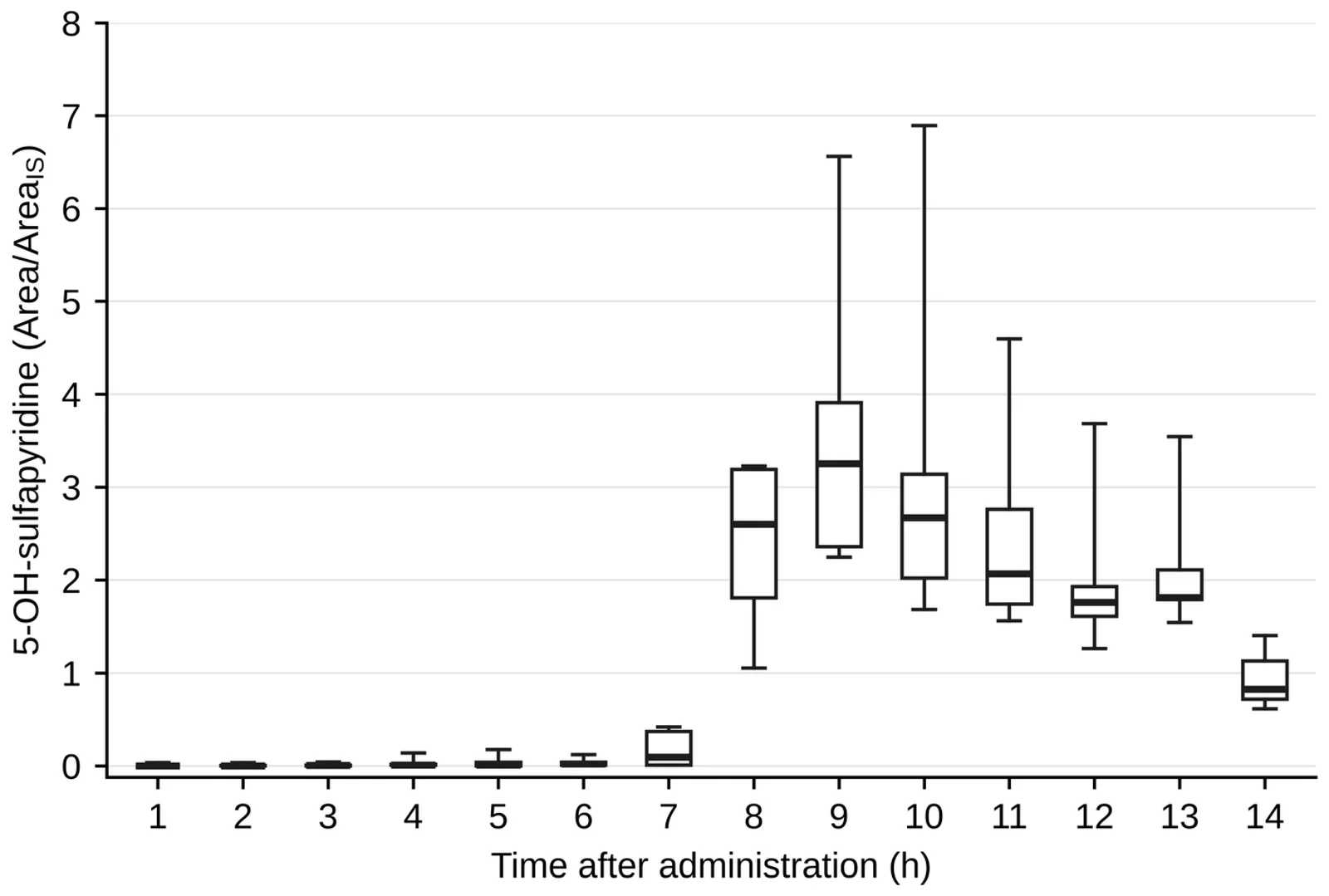
Semi-quantitative 5-hydroxy-sulfapyridine area ratios (Area/Area_IS_) in six healthy adult horses at 14 hourly time points. Data are presented as median (Q1–Q3), with minimum-maximum values. Values are dimensionless and are not comparable with those in Figure 4.

**Figure 4:**
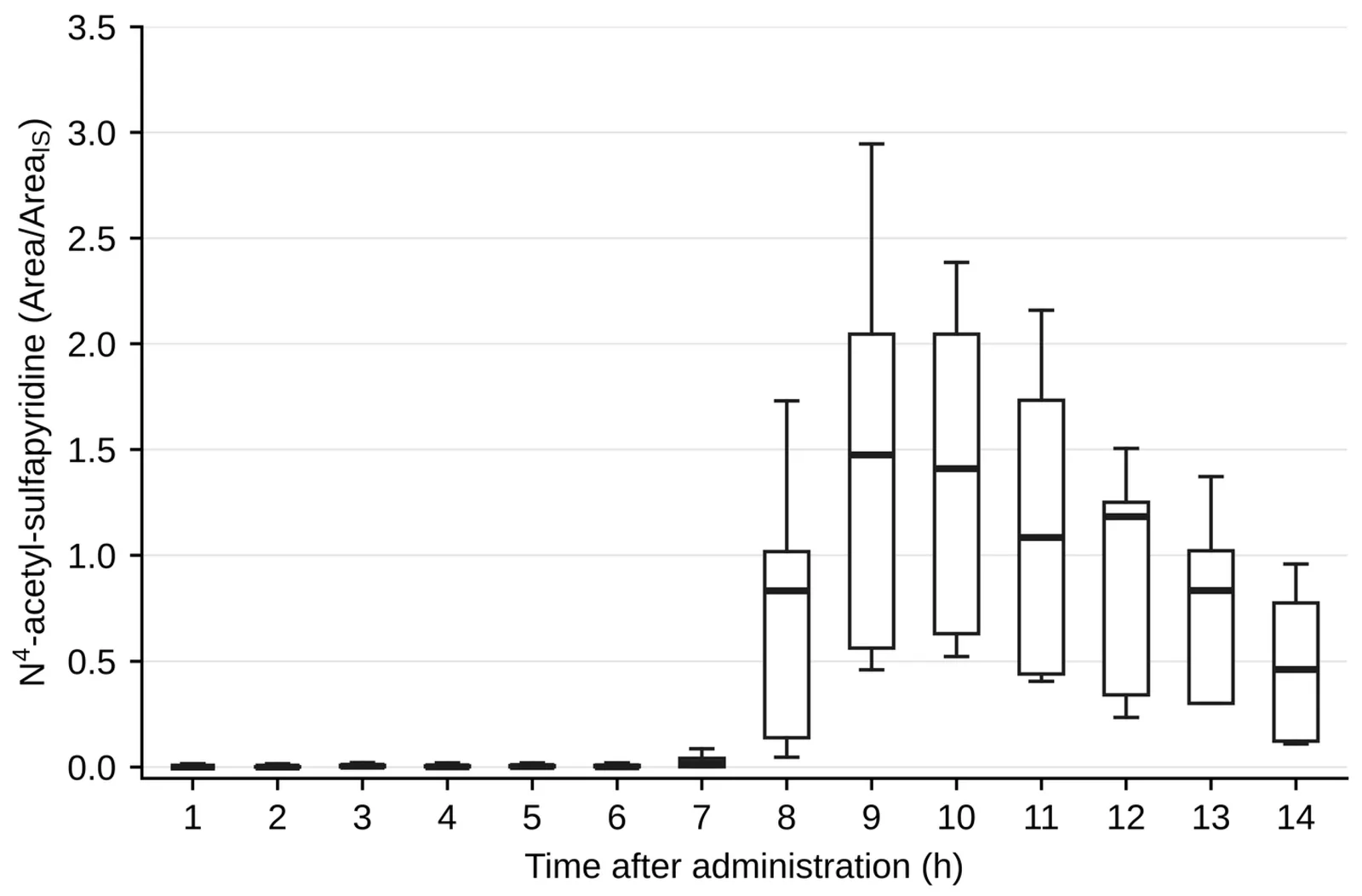
Semi-quantitative N^4^-acetyl-sulfapyridine area ratios (Area/Area_IS_) in six healthy adult horses at 14 hourly time points. Data are presented as median (Q1–Q3), with minimum-maximum values. Values are dimensionless and are not comparable with those in Figure 3.

Both metabolites rose in parallel with sulfapyridine and were present only at trace levels before 7 h. Both ratios increased sharply between 7 and 8 h, peaked at 9 h and then declined, remaining well above baseline at 14 h (Table 1; Figure 3 and Figure 4).

### 3.5. Pharmacokinetic Parameters

Non-compartmental pharmacokinetic parameters are presented in Table 2.

Sulfapyridine T_max_ was 9 h in all six horses. On a molar basis, median C_max_ and AUC_0–14h_ of sulfapyridine were 11.5-fold and 9.9-fold higher, respectively, than those of sulfasalazine (7.2-fold and 6.2-fold on a mass basis).

The temporal distribution of exposure differed markedly between the two compounds. For sulfasalazine, 86.7% of AUC_0–14h_ accumulated before 7 h, whereas 99.6% of sulfapyridine AUC_0–14h_ accumulated after 7 h.

## 4. Discussion

This study provides the first description of sulfasalazine disposition in horses. Three findings are of particular interest: systemic exposure to intact sulfasalazine was low and short lived; sulfapyridine appeared abruptly at 7–8 h and subsequently dominated systemic exposure; and both hepatic metabolites of sulfapyridine appeared in parallel with the parent amine.

### 4.1. Low Systemic Exposure to Intact Sulfasalazine

Median C_max_ for intact sulfasalazine was 386.61 ng/mL, or less than 0.4 µg/mL, following a dose of 16 mg/kg, equivalent to approximately 8 g in a 500 kg horse. By comparison, humans receiving 4 g daily, approximately 57 mg/kg/day in a 70 kg adult, achieve steady state serum sulfasalazine concentrations of approximately 6 µg/mL [1]. The horses in the present study therefore received roughly one third of the human dose on a body weight basis, as a single administration rather than at steady state, and the two figures cannot be compared directly; the comparison is offered only to indicate the order of magnitude. The contrast with a conventional, non-azo-linked sulfonamide in the same species is more informative: oral trimethoprim/sulfadiazine has a reported bioavailability of approximately 58–67% in fasted horses [46]. Sulfasalazine therefore does not behave like a typical orally administered sulfonamide in the horse, which is consistent with its intended pharmacological design.

This is the expected and, from a therapeutic perspective, desirable behaviour of the molecule. Sulfasalazine is designed to function as a poorly absorbed carrier: in humans, only approximately 3–15% of an oral dose is absorbed intact from the proximal gastrointestinal tract [1,4], with the remainder reaching the colon without cleavage. The present data are consistent with similar behaviour in the horse. It should be emphasised, however, that a low plasma concentration of parent drug does not by itself establish that the remainder of the dose reached the hindgut chemically intact; it is equally compatible with incomplete dissolution or with precaecal degradation. The subsequent appearance of sulfapyridine demonstrates that a proportion of the dose did reach an azoreductase-competent bacterial population unchanged, but the size of that proportion cannot be quantified without recovery of sulfasalazine and 5-ASA from luminal contents or faeces. The plateau in parent-drug concentrations between 4 and 7 h, rather than a sharply defined peak, is compatible with slow, ongoing absorption of a small fraction from a large luminal reservoir and with the prolonged gastric retention and progressive small intestinal transit described in horses [31].

The apparent rate of decline of sulfasalazine after 8 h also merits comment. Median concentrations fell from 57.18 ng/mL at 8 h to 1.58 ng/mL at 11 h, corresponding to an apparent half life of well under one hour, which is considerably shorter than values reported in humans. Two considerations apply. First, the decline is calculated here from the median profile rather than from individual terminal slopes, and the values from 11 h onward approach the limit of quantification, so the estimate is imprecise. Second, and more importantly, the profile is input-limited: for a compound of which only a small fraction is absorbed and whose absorption ceases abruptly when the luminal reservoir is cleaved, the observed decline reflects the cessation of absorption superimposed on elimination and cannot be interpreted as a terminal elimination half life. Determination of the true elimination half life would require an intravenous arm and an extended sampling period.

The observed T_max_ also requires interpretation in relation to the median concentration profile, which peaked at 4 h. This apparent discrepancy reflects the method of summarisation rather than a contradiction in the data. T_max_ and C_max_ were determined for each individual horse and then summarised, whereas Figure 1 represents a summary of concentrations at each time point. When individual peaks occur across the 4–6 h interval, the median of the individual T_max_ values does not necessarily coincide with the peak of the median concentration profile. Both measures are reported and should be interpreted separately.

### 4.2. The 7–8 h Transition Reflects Orocaecal Transit

The most striking feature of the dataset is the abrupt transition between 7 and 8 h, during which sulfasalazine concentration decreased approximately five-fold while sulfapyridine concentration increased approximately fifty-fold within a single sampling interval (Figure 1 and Figure 2).

An analytical explanation for this pattern can be reasonably excluded. The transition was observed in every horse, involved four analytes measured in two separate chromatographic channels moving in opposite directions within the same samples, and occurred at a point unrelated to batch or run order. Blank and carryover samples were included in every analytical batch, and sulfapyridine concentrations above the upper limit of quantification were handled as described in Section 2.5. A systematic analytical artefact producing a simultaneous fall in one analyte and a fifty fold rise in another within the same injection is therefore implausible.

Two further lines of evidence support the biological interpretation that this pattern represents the prodrug reaching the fermentative hindgut.

First, this interpretation is consistent with the mechanism of sulfasalazine activation. Liberation of sulfapyridine depends on reduction of the azo bond by bacterial azoreductases, an obligatory step demonstrated by the near complete failure of activation in germ free animals and in patients following colectomy [4,5]. Sulfapyridine therefore cannot appear systemically until the prodrug encounters a sufficiently large bacterial population. In humans, the timing of sulfapyridine appearance is sufficiently reproducible for plasma or salivary concentrations after sulfasalazine administration to be used as a measure of orocaecal transit time [13,14], and the same principle has been applied in dogs [15].

Second, the timing is consistent with published equine transit data. Reported orocaecal transit times in healthy horses range from 5.77 ± 1.67 h using lactose ^13^C-ureide breath testing [29] to a mean of 8.02 h using a wireless capsule [30], with reported caecal arrival half-times of 5.62 ± 1.22 h and a lag phase of 3.24 ± 0.65 h [28]. Passage through the stomach and small intestine averages approximately 5 h, and most digesta reaches the caecum and ventral colon within several hours of feeding [31]. A sulfapyridine appearance time of 7–8 h therefore falls within the range predicted by independent equine transit measurements. Because the observed appearance time additionally incorporates the time required for azoreduction, absorption of released sulfapyridine from the large intestine, and systemic distribution, it should be regarded as an upper bound on orocaecal transit rather than a direct measure of it. The one-hour sampling interval further limits temporal resolution. In addition, the horses were fasted for 12 h before dosing and refed only 6 h afterwards, so the transition time observed under this regime may differ from that in horses on a routine feeding schedule (Section 4.5).

The practical implication is twofold. In the horse, these findings suggest that sulfasalazine behaves as intended: it survives passage through the proximal gastrointestinal tract, reaches the hindgut in a form that remains susceptible to azoreduction, and is activated there. More broadly, the findings raise the possibility that the sulfasalazine method could be adapted as a simple, minimally invasive measure of orocaecal transit in horses, as has been done in humans and dogs. Formal validation against an established technique, and denser sampling around the transition, would be required before such an application could be considered reliable.

It is important to emphasise that azoreductase activity has not been demonstrated directly in equine gastrointestinal contents, and no published study has examined microbial drug biotransformation in this species. The inference that equine hindgut bacteria perform this reduction is based on the present pharmacokinetic findings together with the known taxonomic composition of the equine caecum and colon, which is dominated by Firmicutes and Bacteroidetes and includes the genera *Clostridium*, *Eubacterium*, *Butyrivibrio* and *Bacteroides*, in which azoreductase activity has been demonstrated in other hosts [6,23,24,26]. Direct demonstration, for example by incubating sulfasalazine with equine caecal contents in vitro, would address this remaining uncertainty and represents a logical next step.

### 4.3. Magnitude of Sulfapyridine Exposure

Sulfapyridine dominated systemic exposure (Table 2). Median C_max_ was 7.2 fold higher than that of the parent drug and truncated AUC 6.2 fold greater on a mass basis; because sulfapyridine has a lower molecular weight than sulfasalazine, the corresponding molar differences are larger still, at 11.5 fold and 9.9 fold respectively. The temporal separation was also pronounced: 86.7% of sulfasalazine AUC_0–14h_ accrued before 7 h, whereas 99.6% of sulfapyridine AUC_0–14h_ accrued after 7 h. Thus, a single dose produced two sequential and almost non-overlapping pharmacokinetic phases.

This finding is clinically relevant because sulfapyridine, rather than sulfasalazine or 5-ASA, is the principal determinant of systemic sulfonamide-related adverse effects in humans. Historically, efficacy has been associated with total sulfapyridine concentrations of 20–50 µg/mL and toxicity with concentrations above 50 µg/mL [2]. The median peak sulfapyridine concentration observed in the present study, 2.79 µg/mL, was approximately an order of magnitude below the lower end of that range. This comparison must be interpreted cautiously: the present study evaluated a single dose in a different species at approximately one-third of the human body weight adjusted daily dose, whereas the human thresholds refer to steady state concentrations during chronic treatment. Because the elimination half life of sulfapyridine in the horse is unknown and could not be estimated from the present sampling window, the extent of accumulation that would occur on repeated administration cannot be predicted, and the margin observed after a single dose should not be assumed to persist during chronic dosing.

### 4.4. Metabolite Pattern and Acetylation Capacity

Both hepatic metabolites of sulfapyridine were detectable and their time courses closely paralleled that of the parent amine, with the ratio time curves for both rising from near baseline values at 7 h to peak values at 9 h (Figure 3 and Figure 4). Because both metabolites were assessed semiquantitatively as peak area ratios relative to a common internal standard, and because ionisation efficiency differs between compounds in the absence of determined response factors, the present data support conclusions about the time course of each metabolite but not about their relative abundance. No comparison of the magnitude of the two metabolite signals is therefore drawn from these data.

The existing literature nevertheless allows a prediction as to which pathway should predominate in this species, and it is one that the present study was not designed to test. Hepatic cytosolic acetylation capacity in the horse, measured using p-aminobenzoic acid and isoniazid, is low but detectable and substantially lower than in rabbits and several other species studied [37]. In vivo, N^4^-acetylated sulfadimidine metabolites account for well below 1% of plasma metabolites in horses, whereas hydroxylated derivatives predominate and undergo extensive glucuronidation [38,39]. A profile in which hydroxylation exceeds acetylation would therefore be expected in the horse, and quantitative confirmation of this for sulfapyridine, which will require synthesis or acquisition of certified reference standards for both metabolites, is a clear priority for subsequent work. Equine phase I and phase II biotransformation pathways have been reviewed in detail elsewhere, but arylamine N-acetyltransferase is notably not addressed [47], reflecting the limited information currently available for this pathway in horses.

Comparison with other species is also informative. Dogs lack functional arylamine N-acetyltransferase because NAT genes are absent from the genomes of domestic dogs and the wild canids examined [48]. Horses are clearly different in this respect: N^4^-acetyl-sulfapyridine was detectable and its temporal profile paralleled that of the parent amine, indicating that a functional acetylation pathway is present. Nevertheless, the comparative evidence cited above suggests that acetylation capacity in the horse may be relatively limited. Because the slow acetylator phenotype in humans is associated with greater sulfapyridine exposure and an approximately three fold increase in adverse drug reactions [11,12], it is reasonable to hypothesise that horses may behave functionally as slow acetylators of sulfapyridine. This remains a hypothesis generated by the comparative literature rather than a conclusion supported by the present data. No equine NAT gene has been cloned, sequenced or functionally characterised, and no equine acetylator phenotyping has been reported. Determining whether clinically relevant variation in this pathway exists in horses will require dedicated investigation.

### 4.5. Limitations

Several limitations should be considered when interpreting these findings.

The sample size of six horses was small, and the study was designed primarily as a descriptive characterisation rather than a definitive pharmacokinetic trial. Between animal variability was substantial for several analytes; for example, the interquartile range for sulfapyridine at 7 h extended from 5.43 to 160.30 ng/mL. A study of this size cannot identify the sources of such variability.

The 14 h sampling period represents the most important limitation. Sulfapyridine remained present at 390.82 ng/mL at the final sampling point (Table 1; Figure 2), and the concentration-time curve had clearly not returned to baseline. Consequently, truncated AUC_0–14h_ underestimates total systemic exposure, while terminal half-life and clearance cannot be estimated reliably for any analyte. Conventional criteria require at least three quantifiable concentrations spanning at least one to two terminal half-lives and require the observed AUC to account for at least 80% of AUC_0–∞_ [44,45]. Reported sulfonamide half-lives in horses range from 2.7 to 14.0 h [49], and the human sulfapyridine half-life is 10–15 h depending on acetylator status [1]. A 14-h sampling window is therefore likely to be insufficient for the more slowly eliminated analytes. In addition, the AUC values reported here were calculated from the median concentration profiles rather than as the median of individual AUC values and should therefore be considered approximate summary quantities that carry no measure of between-animal variability. A study extending sampling to 48 or 72 h, with individual non-compartmental analysis, would be required to characterise elimination adequately.

Absolute bioavailability cannot be determined because the study did not include an intravenous treatment arm. The very low aqueous solubility of sulfasalazine makes intravenous comparison technically challenging, although such an approach has been used in dogs [50].

No pre-dose blood sample was collected. The absence of prior sulfonamide exposure was established by history alone, and the assumption of zero concentration at time zero was not verified analytically.

Horses were fasted for 12 h before dosing and hay was withheld until 6 h after administration. This regime differs substantially from routine feeding, and because feeding state was not varied, its effect remains unknown. This is an important limitation because feeding influences both oral drug absorption and gastrointestinal transit in horses [31,32,34,49]. The timing of sulfapyridine appearance in the present study is effectively a measure of transit to the fermentative hindgut and was probably influenced by the prolonged fast and delayed refeeding; both the appearance time and the absorption of intact prodrug may therefore differ in horses fed according to a normal regime.

The metabolites were assessed semi-quantitatively because reference standards were unavailable, which permits description of their time courses but no quantitative statement about their relative or absolute abundance. Neither 5-ASA nor its acetylated metabolite was measured, so the extent of prodrug activation cannot be quantified and no mass balance can be presented.

Finally, the study was conducted only in healthy horses. Gastrointestinal disease, the clinical context in which sulfasalazine might ultimately be considered, can alter both gastrointestinal transit and microbial composition. The disposition observed in the present study may therefore not be directly applicable to clinical patients.

### 4.6. Clinical Perspective

Sulfasalazine is established in the treatment of inflammatory bowel disease in humans and dogs and is used off label in horses [22], but clinical evidence in this species is limited to a single case report [20], and it has not been formally evaluated, including in the context of right dorsal colitis [18]. The present findings provide the first pharmacokinetic basis for considering its use in this species and are broadly encouraging: the prodrug appears to reach the hindgut in an activatable form, sulfapyridine is liberated and absorbed, and the resulting systemic exposure after a single dose is modest by comparison with human data.

Several important cautions remain before clinical use can be considered. The single 16 mg/kg dose used in this study falls within the per-administration range used in dogs (10–30 mg/kg every 8–12 h), but the corresponding canine daily dose is approximately 20–90 mg/kg/day, so the total daily exposure investigated here was considerably lower than that used therapeutically in dogs and lower still than usual human exposure. No dose-ranging study has been performed in horses, and the present data cannot establish an effective dose because 5-ASA, the moiety responsible for local anti-inflammatory activity, was not measured. Measurement of 5-ASA and its acetylated metabolite in intestinal contents or faeces, rather than plasma sulfapyridine alone, would provide more direct information on potential therapeutic activity, and would also permit an estimate of the proportion of the administered dose that is actually activated.

Adverse effects reported in other species also require consideration. Keratoconjunctivitis sicca is a well-recognised consequence of sulfasalazine administration in dogs, was permanent in all but one of thirteen affected animals in one series [51], and has also been reproduced following administration of 5-ASA alone [52], indicating that it cannot be attributed solely to the sulfonamide moiety. Sulfonamide use in horses also has the potential to disturb intestinal microbiota, although repeated trimethoprim/sulfadiazine administration produced only a transient reduction in coliforms without major disruption [53]. Any prospective clinical study of sulfasalazine in horses should therefore include ophthalmic assessment and monitoring of faecal consistency and microbial composition.

## 5. Conclusions

Following nasogastric administration of sulfasalazine at 16 mg/kg to six healthy adult horses, systemic exposure to intact prodrug was low and occurred predominantly during the first seven hours. Sulfapyridine appeared abruptly at 7–8 h and subsequently dominated systemic exposure, exceeding the parent drug by approximately six- to seven-fold in both peak concentration and truncated area under the curve on a mass basis. Both hepatic metabolites of sulfapyridine appeared in parallel with the parent amine and followed a closely similar time course.

The timing of sulfapyridine appearance closely matches published equine orocaecal transit times and supports the conclusion that a proportion of an oral sulfasalazine dose reaches the fermentative hindgut of the horse in a form susceptible to microbial azoreduction and is activated there, consistent with the behaviour for which the molecule was designed. The proportion of the dose so activated cannot be quantified from these data. These findings provide a pharmacokinetic basis for further investigation of sulfasalazine in horses and identify several necessary next steps: extending the sampling period to characterise elimination, including an intravenous arm to determine bioavailability, measuring 5-aminosalicylic acid in intestinal contents to quantify activation, directly demonstrating azoreductase activity in equine hindgut contents, and undertaking dose-ranging studies with appropriate safety monitoring before clinical application is considered.

## Figures and Tables

**Table 1:** Time-dependent changes in serum sulfasalazine and sulfapyridine concentrations and in the semi-quantitative ratios of 5-hydroxy-sulfapyridine and N^4^-acetyl-sulfapyridine, following nasogastric administration of sulfasalazine at 16 mg/kg to six healthy adult horses.

Time Point	Sulfasalazine (ng/mL)	Sulfapyridine (ng/mL)	5-OH-Sulfapyridine (Ratio)	N^4^-Acetyl-Sulfapyridine (Ratio)
1 h	45.88 (27.91–179.82)	<LLOQ (<LLOQ to 1.45)	0.001 (0.000–0.001)	0.000 (0.000–0.000)
2 h	105.64 (41.07–218.77)	1.74 (<LLOQ to 3.08)	0.000 (0.000–0.005)	0.000 (0.000–0.001)
3 h	203.18 (188.74–430.05)	3.82 (2.44–7.27)	0.005 (0.000–0.010)	0.004 (0.001–0.008)
4 h	333.59 (228.31–489.89)	4.78 (2.90–11.50)	0.010 (0.010–0.020)	0.002 (0.001–0.006)
5 h	290.28 (246.36–529.12)	8.49 (3.49–15.83)	0.010 (0.000–0.040)	0.003 (0.001–0.007)
6 h	301.43 (246.89–343.17)	5.26 (4.16–19.81)	0.022 (0.010–0.040)	0.002 (0.001–0.008)
7 h	288.93 (245.74–326.19)	40.03 (5.43–160.30)	0.095 (0.010–0.371)	0.016 (0.002–0.042)
8 h	57.18 (26.43–152.07)	2173.78 (723.71–2394.84)	2.600 (1.808–3.190)	0.832 (0.138–1.017)
9 h	7.80 (4.11–17.23)	2693.60 (2162.64–3176.35)	3.250 (2.360–3.910)	1.474 (0.562–2.045)
10 h	4.32 (2.35–7.62)	1898.75 (1827.57–2109.23)	2.670 (2.020–3.140)	1.409 (0.630–2.046)
11 h	1.58 (<LLOQ to 2.69)	1429.93 (1161.76–1665.11)	2.067 (1.740–2.760)	1.084 (0.439–1.732)
12 h	1.31 (<LLOQ to 3.46)	976.59 (651.51–1220.63)	1.759 (1.610–1.930)	1.183 (0.341–1.250)
13 h	2.39 (1.71–2.63)	781.62 (722.99–1009.97)	1.810 (1.790–2.110)	0.834 (0.301–1.022)
14 h	<LLOQ	390.82 (220.56–470.08)	0.824 (0.720–1.130)	0.461 (0.123–0.775)

Data are presented as median (Q1–Q3); *n* = 6. Sulfasalazine and sulfapyridine were quantified against calibration standards and are expressed in ng/mL; the lower limit of quantification (LLOQ) was 1 ng/mL for both analytes, and concentrations below the LLOQ, which could not be quantified, are reported as <LLOQ. 5-Hydroxy-sulfapyridine and N^4^-acetyl-sulfapyridine were assessed semi-quantitatively and are expressed as dimensionless analyte/internal-standard peak-area ratios (Area/Area_IS_); these values are not concentrations, are not comparable between the two metabolites, and are not comparable with other studies.

**Table 2:** Pharmacokinetic parameters of sulfasalazine, sulfapyridine, 5-hydroxy-sulfapyridine and N^4^-acetyl-sulfapyridine in six healthy adult horses following nasogastric administration of sulfasalazine at 16 mg/kg.

Parameter	Sulfasalazine	Sulfapyridine	5-OH-Sulfapyridine	N^4^-Acetyl-Sulfapyridine
C_max_	386.61 ng/mL (330.44–504.35)	2794.00 ng/mL (2381.87–3146.14)	3.45 (2.97–3.86)	1.54 (0.83–1.95)
T_max_ (h) ^b^	5.5 (4.25–6.0)	9.0 (9.0–9.0)	9.0 (8.25–9.0)	9.5 (9.0–10.0)
AUC_0–14h_ ^a^	1643.6 ng·h/mL	10,214.6 ng·h/mL	14.71 h	7.07 h

C_max_, maximum observed concentration (sulfasalazine, sulfapyridine) or maximum observed area ratio (metabolites); T_max_, time to C_max_; AUC_0–14h_, area under the concentration-time curve (sulfasalazine, sulfapyridine) or under the ratio-time curve (metabolites) from 0 to 14 h. C_max_ and T_max_ are presented as median (Q1–Q3) of individual values. ^a^ AUC_0–14h_ was calculated by the linear trapezoidal rule from the median concentration-time profiles, with the concentration at time zero assumed to be zero; it is therefore a single derived value rather than a median of individual estimates, and no interquartile range is given. Because sulfapyridine had not returned to baseline at 14 h, AUC_0–14h_ underestimates total exposure, and AUC_0–∞_ is not reported (see Section 4.5). ^b^ T_max_ values were read directly from the hourly sampling times; medians falling between sampling times (e.g., 5.5 h) arise from the even number of animals (*n* = 6).

## Data Availability

The data presented in this study are available on request from the corresponding author. The digitised data and plotting code used to prepare the figures are also available on request from the corresponding author.

## Supplementary Materials

The following supporting information can be downloaded at: https://www.mdpi.com/article/10.3390/ani16213530/s1, Figure S1: Individual serum sulfasalazine concentration-time profiles in six healthy adult horses following nasogastric administration of sulfasalazine at 16 mg/kg; each line represents one horse.
